# Short Assay Design for Micronucleus Detection in Human Lymphocytes

**DOI:** 10.1155/2021/2322257

**Published:** 2021-09-11

**Authors:** Guido Rincón, Claudia Sánchez

**Affiliations:** Applied Science, Antonio Nariño University, Bogotá, Colombia

## Abstract

There has been a constant need to develop new and faster cytogenetic assays to measure the instability induced by genotoxic agents in the field of cytogenetic research, an example of which is the micronucleus assay. Micronuclei are fragments or complete chromosomes that remain in the cytoplasm during mitosis. With their high sensitivity and specificity detection, their presence can indicate environmental and occupational genotoxic effects. However, the prolonged periods of cell incubation this assay necessitates are costly and extensive. Hence, it is essential to develop an improved assay that can achieve standardization by being reproducible in practice. The standard protocol for the detection of micronuclei in lymphocytes uses a total assay time of 72 hours. Theoretically, it is possible to reduce the incubation period, and consequently, the total assay time, considering a lymphocyte, completes its mitosis in 24 hours. This study, after careful review of literature, proposes an experimental design to reduce the incubation period and demonstrates its usefulness in practice through the design of a collaborative trial.

## 1. Introduction

In 2011, the International Atomic Energy Agency (IAEA) recommended the use of four cytogenetic techniques to assess radiological emergencies and preparedness: dicentric analysis (DCA), micronucleus (MN) assay, fluorescent *in situ* hybridization (FISH), and premature condensation of chromosomes (PCC) [1]. In this study, we focused on the micronucleus assay. Micronuclei measure genotoxic damage from exposure to ionizing radiation by detecting chromosomal fragments. However, this technique requires long incubation periods and monitoring of *in vitro* cultures for more than 72 hours. Such a long wait for assay results does not help in critical situations of possible terrorist attacks or radiological emergencies. Hence, it is necessary to reduce the incubation period to be able to quantify the changes caused by exposure to radiation and DNA damage in the shortest possible time.

The International Agency for Research on Cancer (IARC) classifies ionizing radiation in humans as a cancer-inducing agent. Studies with workers occupationally exposed to this type radiation [2, 3] demonstrated a significant association between low dose exposure and cancer development [4].

The use of MN assay to study micronuclei as a predictive marker, or for biomonitoring purposes, offers high sensitivity and specificity of diagnosis in genotoxic and cytotoxic damage [5], especially in medical personnel receiving frequent occupational radiation, such as intervention cardiologists, technologists, and radiologists [6–8]. The MN assay also predicts low radiation exposure [9] and its relationship with cancer induction. However, the incubation period is too long [10], and the correlation of variables has been a constant challenge for standardization the optimal operation of the technique. Additionally, reproducibility in normal clinical practice that leads to a rapid diagnosis is also a challenge that needs addressing [11].

During the incubation period, cytochalasin B (Cyt-B) is added to stop cytokinesis and achieve binucleated cells (BN), which express micronuclei in lymphocytes [12], and complete their division every 24 hours [13, 14]. However, they can be lost during mitosis [12]. Hence, it is necessary to use novel or faster exposure techniques [15].

Some studies using organ cells, such as those of the liver, have proven the possibility of reducing the incubation periods of micronuclei [16] and describe the development of MN assay protocols that requires 48 hours of treatment with Cyt-B.

Other studies, too, have concluded the need to shorten the time it takes for MN assay completion, especially in the case of triage radiation biodosimetry, where a high number of possible victims need results as quickly as possible [17]. However, an interlaboratory comparison exercise is required to validate changes in the total time of the assay, e.g., the one performed by the RENEB (European Network of biological dosimetry) [18]. Alternatively, an analysis of blind samples in different laboratories [19] is needed, with the use of varied incubation periods, to obtain interlaboratory reproducibility. Modification of the protocol time has been tested in hematopoietic cells, too, highlighting the need for a deeper characterization of the sensitivities and cytostatic subtypes analyzed over time [20].

To summarize, current research to validate the adjustment in the shorter assay time still lags behind, since additional evidence is needed to prove that a shorter time can be applied to assays using human lymphocytes and that they have the same diagnostic predictive power. It is essential to note that lymphocytes are the main cells of the immune response that transmit heredity and have a high half-life. Therefore, additional research is necessary to validate an accorded standard operating procedure of the MN assay [11, 21] for human lymphocytes.

The standard micronucleus protocol uses full *ex vivo* blood and requires an incubation time of 44-72 hours, which can be minimized [22]. This is mentioned by a multiparametric study that concludes that due to time limitations, it is necessary to perform analysis in different laboratories, in order to have prolonged incubation times [23]. The standardized operating procedure (SOP) can be adjusted, but each change requires several rounds of validation, incurring large reagent and personnel costs (according to the protocol described for Fenech and evaluated internationally) [24].

Besides, experimental tests are a necessity, where different incubation periods are tested in collaboration with other laboratories and the predictive power of the test (MN); its efficacy and probability are demonstrated. Mitogenic stimulation during early and late incubation periods can also be studied in such experiments [25].

## 2. Theoretical Framework

The presence of micronuclei indicates lagging DNA or incomplete DNA synthesis, which can be caused due to exposure of cells to toxic agents such as ionizing radiation. The frequency of lagging or incomplete DNA is called micronuclei; its presentation indicates the adaptation of cells to toxic agents such as ionizing radiation and is important according to the theory of the human genome [26]. Among cells that express micronuclei are lymphocytes and are radiosensitive since their differentiation involves several divisions (“law of Bergonié and Tribondeau,” 1906). The standard protocol for the detection of micronuclei in lymphocytes currently uses an incubation period of 72 hours [24], and it is theoretically possible to decrease the incubation time considering a lymphocyte completes its mitosis in 24 hours.

To carry out this research, a minimum of three laboratories is needed to develop the modification of the incubation period and perform statistical analysis. Subsequently, joint research or standardization is required in the development of the technical proposal and validation of a short protocol of 48-56 hours, against the standard method of 72 hours. It is also necessary to explore ways in which the mitogenic response can be accelerated [11, 21] and verify the optimal laboratory conditions needed such as ISO 17025, 5725 calibration, testing, and the sterility conditions.

## 3. General Methodology

Validation studies (collaborative trials) are used to characterize and standardize methods between laboratories [27]. Here, we have designed a collaborative study between laboratories [28, 29], to demonstrate that the incubation period for lymphocytes in the standard operating procedure in the MN assay can be set at 24 hours, without altering the final count of micronuclei and its diagnostic power.

This type of collaborative study provides great strength to demonstrate if the variable time change works entirely, because it takes into account features like performance study, conduct, compliance, and behavior [30]. Additionally, the study

- analyzes the reproducibility of the micronuclei score between times
- evaluates the effect of changing the standard incubation period with respect to the number of micronuclei found
- studies the role of the two incubation times in the final result by adding Cyt-B and the nuclear division index in cultures of delivered blind samples [31]

This collaborative study will begin by sending an invitation letter to laboratories that have published studies using the MN assay on human lymphocytes, those who have been consulted in indexed journal databases, and those who wish to participate [32]. After acceptance by a minimum of three or five laboratories according to Horwitz and Miller et al. [30, 33], a questionnaire will be sent to obtain basic information on the data available in each laboratory: address, laboratory protocol, incubation time, scoring criteria, incubation temperatures, centrifugation protocols, control variables, and quality control procedures.

### 3.1. Ethical Considerations

This study will be aimed at being risk-free. The privacy and confidentiality of the participating laboratories will be maintained with a code and number. Participating subjects will sign a consent authorizing the process. The results of the study will be sent to each participating laboratory after the study analysis. There are no risks or benefits associated with handling samples and/or specimens for participating laboratories, and there will be no effects on the subjects participating in the research. The information obtained and the results generated will be shared with the participants of the laboratories.

### 3.2. Validity

This study will be aimed at determining if by blocking cytokinesis at 24 hours, lymphocytes will exhibit the same number of cultured micronuclei as those cultured for 44 hours. Therefore, the selected variable of the SOP MN to evaluate with the collaborative study is “incubation period” [31, 34].

Collaborative studies must be validated so that they can be professionally evaluated. The proposed protocol incorporates changes to the first international protocol described by Fenech [24].

Demonstration of the validity of the time change [35] should generate interest in organizations such as the International Atomic Energy Agency, Retrospective Biological Physical Dosimetry Network, and International Organization for Standardization (ISO 17099-19238). In addition, this study should assist in the analysis of biological effects in the event of nuclear and radiological emergencies [36–38].

**Figure 1:**
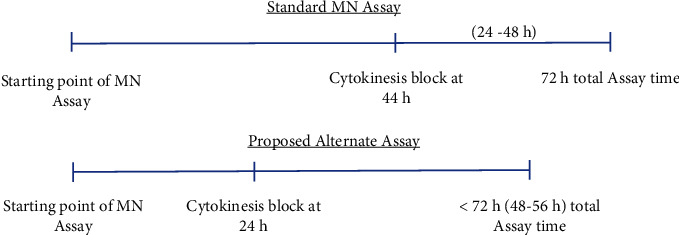
Scheme showing the beginning and the end of the original vs. proposed assay.

To validate the proposed time change, the physical conditions of the variables must be guaranteed. These included centrifugation conditions and temperature so that the analysis and the count of cells that have completed their mitosis from start to finish are reproducible and that they express micronuclei in 24 and 44 hours, respectively (Figure 1) [31, 34].

This study will take into account the theories to improve the odds of cell cycle response and the conclusions of studies suggesting the need for faster techniques and diagnostics [11].

The reliability of the collaborative study design will be verified when similar results are obtained between the participating laboratories and similar results are produced in the same sample analyzed to determine the performance of each laboratory and establish comparisons between laboratories.

### 3.3. Sampling

For these collaborative trials, laboratories that meet the minimum requirements of a comparison study in accordance with ISO 5725 and who wish to participate voluntarily are identified, in addition to having experience in the analysis of micronuclei and studies of prevalence or incidence of micronuclei in peripheral blood to achieve validity of the results. The inclusion criteria include laboratories with experience of at least 3 years in cytogenetic techniques, specifically in micronuclei.

The exclusion criteria include laboratories that refuse to participate voluntarily.

#### 3.3.1. Number of Replicates

For the purpose of design and statistical analysis, four test samples identical to each other but differing slightly in micronucleus count (e.g., <1%-5%) will be used in each laboratory [30]. Each sample must be analyzed once.

### 3.4. Combination of Blinds and Replicas

#### 3.4.1. Blind Replica

Identical blind replicas will be sent to each laboratory, or identical nonblind replicas can be used.

#### 3.4.2. Known Replicate

For each laboratory, known replicates will be sent (2 or more analyses of test portions of the same sample) [30].

Blind and known samples are part of the individual analysis.

A nonprobabilistic sampling will be done (there is no previous data) for the first time and will take into account type I and II errors.

Type I error, also called alpha type error (*α*) or false positive, is the error that is committed when the investigator rejects the null hypothesis (H_0_) to be true in the experiment. It is equivalent to finding a false positive result, since the investigator concludes that there is a difference between the hypotheses when there is actually no difference. The error type II, beta error (*β*) or false negative, is equivalent to not finding differences that exist.

### 3.5. Data

Initially, two samples will be sent; however, four blind samples can be used, taking into account the budget to demonstrate the proposed time change. Each laboratory performs the same number of experiments and applies the two techniques: cytokinesis block at 44 hours (process 1) and cytokinesis block at 24 hours (process 2). The data obtained are micronucleus counts. The results are expressed in the number of micronuclei per 1000 binucleated cells in both processes, taking into account the scoring criteria according to HUMN Project [39]. Valid data for each sample will be sent to the laboratory. Valid data are those that would be reported as a result of normal laboratory analysis (micronucleus count in each of the samples). The requirements of acceptance are that the results should not show more than 10% variation, taking into account the final count, the variability between laboratories, and the reference laboratory. Validation will be done by a reference laboratory invited to participate in American or European dosimetry networks.

### 3.6. Analysis

Three days after delivery of the samples, the results of each laboratory will be collected and the statistical analysis calculated if the

- amount of micronuclei is less than the reference value of the standard MN assay
- number of micronuclei is equal to the reference value of the standard MN assay
- number of micronuclei is greater than the reference value of the standard MN assay

#### 3.6.1. Statistical Analysis

The results of the valid data will be those reported as a result of the analysis of each laboratory.

To verify that both experiments are the same (44 h of process 1 vs. 24 h of process 2), the hypotheses are first proposed:

- H_0_ : *μ*_1_ = *μ*_2_
- H_a_ : *μ*_1_ ≠ *μ*_2_

The null hypothesis (H_0_) says that the mean of process 1 is equal to that of process 2.

The alternative hypothesis (H_a_) says that the mean of process 1 is different from that of process 2 (our objective is to not reject the null hypothesis). Verification of the hypotheses is done in two ways: parametric/nonparametric. Normality is verified taking into account the following:

- H_0_: data shows normal distribution
- H_a_: data does not show normal distribution

The distribution of data will dictate the use of parametric or nonparametric tests.

If the distribution is normal, a parametric test such as the “Student *t*-test” is used to compare mean values. If the distribution is not normal, a nonparametric test such as the “Mann-Whitney *U* test” is used to compare median values. The normality of the variable “count” is then evaluated, and if it is met, the data is analyzed using parametric tests such as the *t*-test.

The test calculates the mean for each process. Its distribution must be 0 or very close, and it uses confidence intervals to show that both processes are equal. But if the difference has values far from 0, the difference is significant; i.e., *μ*_1_ − *μ*_2_ is different from *μ*_2_ − *μ*_1_.

The data does not always have a normal distribution. If this assumption is not fulfilled and median values are compared but not means using the Mann-Whitney *U* test, the hypothesis changes from comparing means to medians:

- H_0_ : *Me*_1_ = *Me*_2_
- H_a_ : *Me*_1_ ≠ *Me*_2_

Box and whisker plots can be used to display the results, and the process should be repeated by the 3 or 5 laboratories. The graphics visually allow to understand the equalities or differences of the data according to the result of the statistical test. To prove that the micronucleus count is the same in the 5 participating laboratories, ANOVA (Analysis of Variance) can be performed if the data is normal. In the case of data that is not normal, the Kruskal-Wallis test (KS) can be performed.

## 4. Final Thoughts

The standard MN assay protocol requires a culture period of 72 hours for completion. Theoretically, it is possible to shorten the period to 48-56 hours if cytochalasin B is added 24 hours after the start of the culture, if the experimental conditions are maintained [11]. This is the time it takes for a hematopoietic cell to grow and divide [14] during which micronuclei appear. Reduction of the incubation period can lead to similar results as well, since there are enough lymphocytes that have completed their nuclear division and which can be detected.

The conditions of the proposed modification will be verified *in vitro* and compared with the standard protocol, which can be used as one of the most reliable, well-established, and feasible genotoxicity tests [39], being useful even as a valid marker for the prediction of cancer risk in humans. The design of such a shortened standardized operating procedure for micronucleus detection increases the response capacity to meet the care needs of massive or large-scale victims [19]. However, when monitoring populations, factors such as age, sex, body mass index, history of family cancer, and lifestyle (smoking habit, alcohol consumption, exposure to medications and diagnostic radiation, and physical activity) should be taken into account. Additionally, we must consider other seasonal variations, such as sampling time, sampling period, and different meteorological parameters that can influence the results obtained, considering that the human population is increasingly exposed to environmental factors that affect the frequency of biomarkers [40].

It also supports recent literature suggesting that, theoretically, the culture time can be reduced to obtain similar results (Fenech), considering there is loss of damaged cells during cell culture before Cyt-B addition [41].

Decreasing incubation time in the collaborative trial emphasizes the importance of a validation study to examine the experimental variables associated with micronucleus performance and frequency in human lymphocytes, improving the diagnostic value and reducing staff costs. Furthermore, a shorter test will improve the ability to respond to emergencies, leaks, triage, and decision-making for the staff in charge.

The techniques proposed by IAEA need to be improved in response to large-scale related events such as leaks, accidents, and/or terrorist attacks using short incubation times. In the future, after a sufficient number of such collaborative trials are carried out, their results are likely to be significantly different from those published in the literature. For biodosimetry networks, such a collaborative work is a useful tool that ensures the standardization of techniques to be implemented in the future and enhances the diagnostic utility by proposing its use in inter-laboratory networks. This also leads to the optimization of resources, both inside and outside the experimental environment.
